# Advancing the Evidence on Glabellar Contraction Patterns—A Prospective Multicenter Study on Its Clinical Relevance for Glabellar Neuromodulator Injections

**DOI:** 10.1111/jocd.70911

**Published:** 2026-05-13

**Authors:** Marcelo Germani, Victor R. M. Munoz‐Lora, Adriana M. Geroldo, Helen Hayashida, Lucas Inácio, Samantha Vitale, Vanessa Bulhões, Vanessa Thiesen, Kristina Davidovic, Sergio Escobar, Rod Rodrich, Michael H. Gold, Carlos Bravo, Sebastian Cotofana

**Affiliations:** ^1^ Department of Biological Sciences, Bauru School of Dentistry University of São Paulo Bauru Brazil; ^2^ Department of Facial Aesthetics Guarulhos University São Paulo Guarulhos Brazil; ^3^ HOF Pro Academy Rio Verde Goiás Brazil; ^4^ Private Practice Paraná Brazil; ^5^ Private Practice Joinville Santa Catarina Brazil; ^6^ Private Practice São Paulo Brazil; ^7^ Private Practice Salvador Bahia Brazil; ^8^ Private Practice Florianópolis Santa Catarina Brazil; ^9^ Center for Radiology and Magnetic Resonance Imaging University Clinical Center of Serbia Belgrade Serbia; ^10^ Faculty of Medicine University of Belgrade Belgrade Serbia; ^11^ Private Practice Buenos Aires Argentina; ^12^ Dallas Plastic Surgery Institute Dallas Texas USA; ^13^ Department of Dermatology Gold Skin Care Center Nashville Tennessee USA; ^14^ Private Practice San Jose Costa Rica; ^15^ Department of Plastic Surgery Vanderbilt University Medical Center Nashville Tennessee USA; ^16^ Centre for Cutaneous Research, Blizard Institute Queen Mary University of London London UK; ^17^ Department of Plastic and Reconstructive Surgery Guangdong Second Provincial General Hospital Guangzhou Guangdong Province China

**Keywords:** botulinum toxin type A, facial anatomy, glabellar lines, injection technique, neuromodulators

## Abstract

**Background:**

Glabellar neuromodulator injections are frequently guided by skin surface contraction patterns, assuming that visible rhytids reflect underlying muscular anatomy. However, emerging anatomical and imaging evidence has questioned the clinical relevance of pattern‐based treatment adaptations.

**Objective:**

To prospectively evaluate whether different glabellar contraction patterns influence clinical and patient‐reported outcomes when a standardized, anatomy‐based, FDA‐approved 5‐point injection algorithm is applied.

**Methods:**

This prospective, multicenter clinical study enrolled 125 patients treated across five independent centers. Patients were classified into five previously described glabellar contraction patterns (V‐shape, U‐shape, converging arrows, omega, and inverted omega) based on standardized photographs during maximal frowning. All patients received neuromodulator injections following the same standardized 5‐point technique using either onabotulinumtoxinA or abobotulinumtoxinA with dose equivalence. Clinical outcomes were assessed using the Glabellar Line Severity Scale (GLSS) at baseline, 15 days, and 90 days, while patient‐reported outcomes were evaluated with the Global Aesthetic Improvement Scale (GAIS) at 90 days. Statistical analyses included non‐parametric group comparisons and multivariate ordinal logistic regression.

**Results:**

A total of 119 patients completed the study. A significant improvement in GLSS was observed at 15 days (*p* < 0.001), followed by partial regression at 90 days (*p* < 0.001 vs. baseline). No statistically significant differences in GLSS or GAIS were identified between contraction pattern groups at any time point (all *p* > 0.05). Multivariate analysis demonstrated that age and body mass index significantly influenced treatment outcomes, whereas glabellar contraction patterns and Fitzpatrick skin type did not. Mild, transient local adverse events were observed and resolved spontaneously.

**Conclusion:**

Glabellar contraction patterns do not significantly affect clinical or patient‐reported outcomes when neuromodulator injections are performed using a standardized, anatomy‐based 5‐point technique. These findings support prioritizing muscular anatomy and patient‐specific biological factors over surface rhytid patterns in glabellar neuromodulation.

## 1 Introduction

Neuromodulator injections remain the most frequently performed minimally invasive aesthetic procedure according to the annual report of the American Society of Plastic Surgeons [1]. Of the various facial regions that can be addressed with neuromodulators, the glabella is the most often targeted facial region, reflecting directly on the FDA approved clinical indications of neuromodulator manufacturers in the US [2].

For targeting the glabella, a 5‐point injection algorithm has been approved in 2002, providing guidance for intramuscular injections of glabellar muscles. In 2012, de Almeida et al. proposed to utilize individual skin surface wrinkle configurations during maximal frowning to adjust the approved 5‐point injection algorithm to optimize clinical results [3, 4]. According to this approach, surface anatomy instead of muscular anatomy would guide clinical practice when administering neuromodulators. Due to its clinical ease, this approach has been widely disseminated in clinical practice and educational settings.

However, subsequent investigations have raised questions regarding the clinical relevance of the skin surface‐derived injection algorithm as proposed by de Almeida et al. [5, 6, 7], observational and clinical studies conducted in different populations have reported inconsistent outcomes when treatment strategies were adapted according to the proposed glabellar patterns, suggesting that visible skin rhytids may not reliably reflect the underlying muscular anatomy [6, 8]. A recent magnetic resonance imaging–based study demonstrated no significant differences in the morphology, thickness, or spatial arrangement of the glabellar muscles when stratified by glabellar contraction pattern, further challenging the anatomical and thus clinical rationale of surface‐based treatment modifications [9].

To directly investigate the clinical merit of glabellar contraction patterns, Germani et al. [8] compared the novel 3‐point glabellar injection technique across different contraction patterns in a retrospective study design. The authors reported equally good clinical outcomes independent of the skin rhytid pattern, thereby challenging the usefulness of glabellar contraction patterns. However, no study to date has compared the clinical merit of the 5‐point injection technique across different glabellar contraction patterns in a prospective clinical study design. Two possible outcomes could be expected from such an investigation:

Hypothesis 1 *There is a difference in the clinical outcome between patients with different glabellar contraction patterns resulting in the need to adjust for skin surface rhytids during glabellar neuromodulator treatments (as suggested de Almeida et al.)*.

Hypothesis 2 *There is no difference in the clinical outcome between patients with different glabellar contraction patterns resulting in the confirmation of the FDA approved injection algorithm for glabellar neuromodulator treatments*.

To investigate the above formulated hypotheses, the present multicentric, clinical, prospective study was conducted. It is hoped that the results of this investigation will clarify if the use of glabellar contraction patterns is justified in a clinical scenario to deviate from the FAD approved injection algorithm.

## 2 Material and Methods

### 2.1 Study Design

This study was designed as a prospective, multicenter clinical investigation of glabellar injections conducted between May and September 2025 across five independent centers. It received ethical approval from the Research Ethics Committee of the “Hospital de Reabilitação de Anomalias Craniofaciais da Universidade de São Paulo—HRAC/USP” under the protocol number CAAE—88794525.6.0000.5441. All patients provided written informed consent prior to their inclusion for the use of their demographic and clinical data for medical research purposes. The treatments performed (glabellar injections with neuromodulators) adhered to the departmental standards of care and to the guidelines of Good Clinical Practice.

### 2.2 Study Sample

A total of 125 Brazilian patients (25 from each of the five participating Brazilian centers) were prospectively enrolled. The study population was multi‐ethnic, comprising individuals with moderate to very severe glabellar lines (Table 1). No additional inclusion criteria were imposed to maintain a community‐based approach and reflect real‐world clinical practice.

**TABLE 1:** Demographic characterization of the study sample.

Demographics	Value
Participants	119
Male	12
Female	107
Age	40.4 (±10.50)
BMI	26.60 (±4.90)
GLSS	2.00 (IQR = 1.00)
Group V‐shape	16
Age	41.5 (±13.61)
BMI	25.67 (±4.42)
GLSS	2.00 (IQR = 1.00)
Group U‐shape	40
Age	38.7 (±10.00)
BMI	26.20 (±4.33)
GLSS	2.00 (IQR = 0.250)
Group converging arrows	36
Age	40.19 (±10.04)
BMI	27.54 (±5.93)
GLSS	2.00 (IQR = 2.00)
Inverted omega	13
Age	40.8 (±9.12)
BMI	25.94 (±3.92)
GLSS	2.00 (IQR = 1.00)
Omega	14
Age	44.29 (±10.56)
BMI	26.63 (±4.78)
GLSS	3.00 (IQR = 1)

*Note:* Comparisons among groups according to the glabellar contraction pattern (V‐shape, U‐shape, converging arrows, inverted omega, and omega) were performed using one‐way ANOVA for age and body mass index (BMI), and the Kruskal–Wallis test for glabellar line severity score (GLSS). No statistically significant differences were observed in any of the comparisons. Data are expressed as frequency counts for sex distribution, mean and one standard deviation (±SD) for age and BMI, and median with interquartile range (IQR) for GLSS.

Exclusion criteria comprised the presence of neuromuscular disorders, coagulation abnormalities, known hypersensitivity to botulinum toxin or any of its components, active infections in the injection area, pregnancy or breastfeeding, a history of facial cosmetic surgery, or receipt of any aesthetic treatment, including neuromodulators, within the 12 months preceding enrollment.

### 2.3 Glabellar Contraction Patterns

**FIGURE 1:**
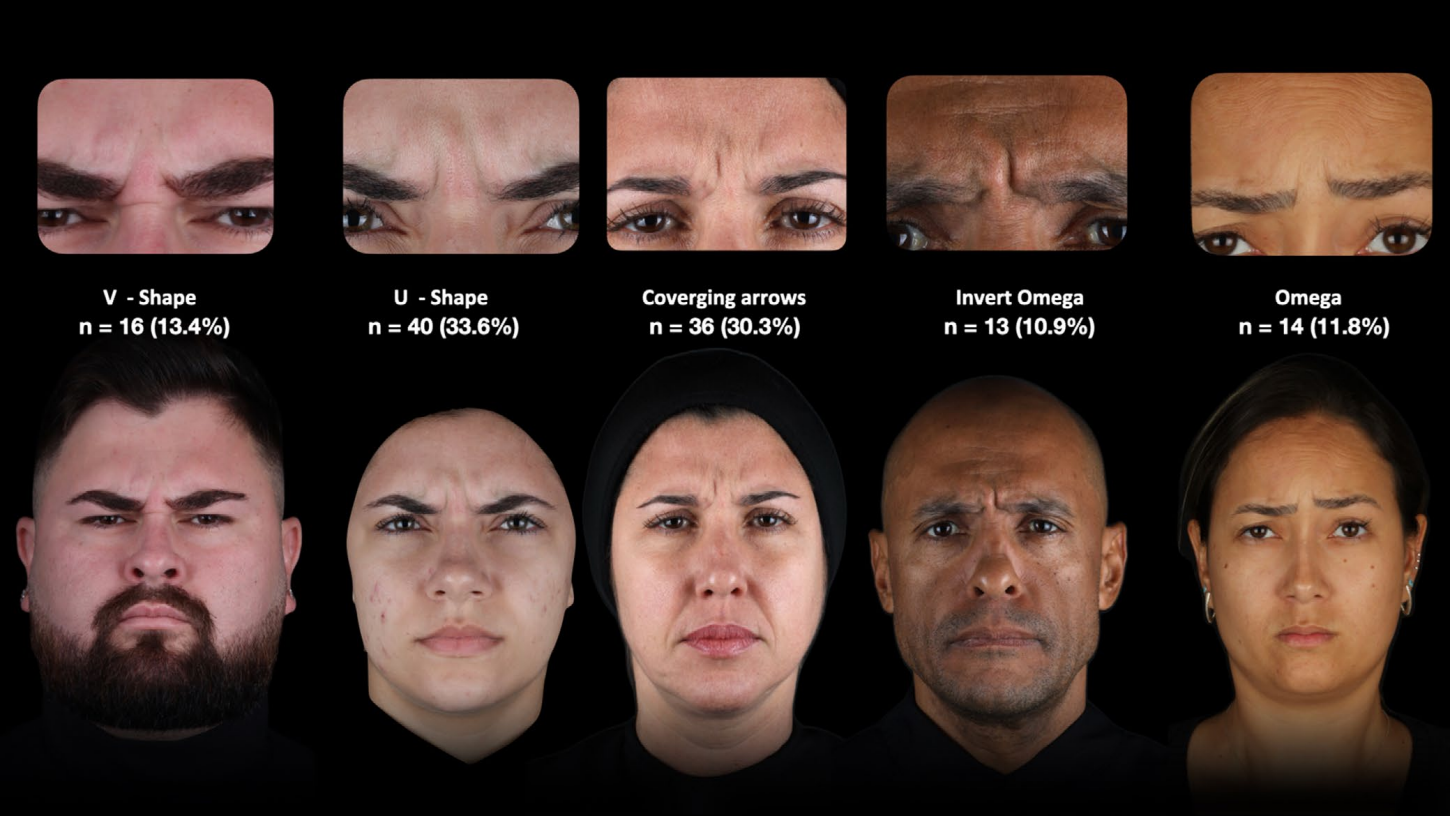
Standardized photographs illustrating the five previously described glabellar contraction types during maximal frown.

To determine the skin rhytid pattern, patients were asked to perform a maximal frown, engaging the procerus (PM), corrugator supercilii (CSM), orbicularis oculi (OOM), and frontalis (FM) muscles. The resulting dynamic skin rhytids were classified into one of five previously suggested glabellar contraction patterns: U‐shape, V‐shape, Converging Arrows, Omega, and Inverted Omega (Figure 1), as previously described in the literature [3].

Classification was based on standardized frontal 2D photographs and was evaluated by two independent injectors, both with more than five years of clinical experience in facial aesthetics. In cases of disagreement, a third senior evaluator decided the final categorization. This protocol ensured a consistent and reliable classification of glabellar contraction types.

### 2.4 Injection Technique

All injections were performed in a single session across the five participating centers by experienced practitioners, each with more than five years of clinical expertise in facial neuromodulator treatments. Patients were treated with either onabotulinumtoxinA (Botox, Allergan Inc., Abbie, Irvine, CA, USA) with each vial containing 100 IU (= international units) reconstituted in 1.0 cc of sterile saline; or abobotulinumtoxinA (Dysport, Galderma SA, Upsala, Sweden) with each vial containing 500 s.U (= Speywood units) reconstituted in 2.0 cc of sterile saline. All products were prepared immediately before application. To ensure dose equivalence, a conversion ratio of 1:2.5 (ona: abo) was applied.

The skin surface was disinfected with 2% chlorhexidine, and topical anesthesia (lidocaine 4%, Dermomax, Aché Laboratórios, Sao Paolo, Brazil) was applied prior to injection. Treatments were carried out with 31G/6 mm syringes (Becton Dickinson, Franklin Lakes, NJ, USA).

**FIGURE 2:**
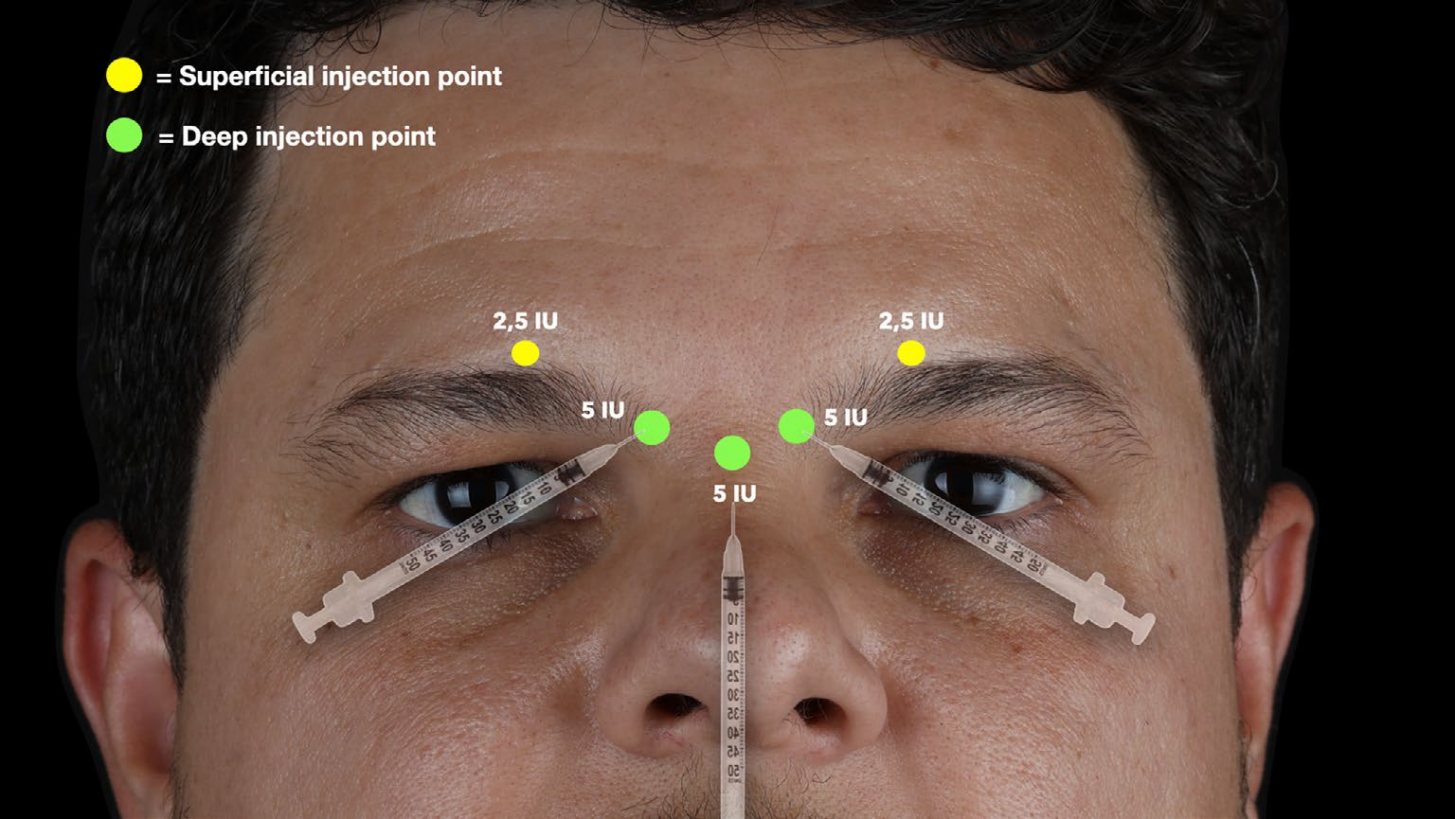
Illustration of the injection technique used in all patients. Two deep injections were placed at the origin of each corrugator supercilii muscle (5 U each), two more superficial injections at their insertion (2.5 U each), and one deep injection at the origin of the procerus muscle (5 U), totaling 20 U of onabotulinumtoxinA or 50 sU of abobotulinumtoxinA per patient.

All patients were treated according to a standardized five‐point injection protocol (Figure 2):

– Corrugator supercilii muscle: Deep, medial supraorbital ridge.
– Procerus muscle: Deep, midline at the level of the medial canthus.
– Orbicularis oculi muscle: Superficial, medial third of the eyebrow at upper margin of eyebrow cilia (this injection is also referred to as the lateral corrugator supercilii muscle injection).

For patients treated with onabotulinumtoxinA, the dose distribution consisted of 5 IU at each corrugator origin, 2.5 IU at each corrugator insertion, and 5 IU at the procerus origin, totaling 20 IU per patient. For patients treated with abobotulinumtoxinA, the equivalent doses were applied, with 12.5 s.U at each corrugator origin, 6.25 s.U at each corrugator insertion, and 12.5 s.U at the procerus origin, totaling 50 s.U per patient.

No additional botulinum toxin treatments were administered to other facial regions during the study period.

### 2.5 Outcomes

Clinical efficacy was assessed with both objective and subjective instruments. The Glabellar Line Severity Scale (GLSS) was applied at baseline, 15 days, and 90 days post‐treatment to grade the severity of dynamic glabellar lines during maximal frowning. This validated 5‐point ordinal scale ranges from 0 = no lines, 1 = mild lines, 2 = moderate lines, 3 = severe lines, to 4 = very severe lines, providing an objective measure of wrinkle severity [10].

Subjective improvement was evaluated using the Global Aesthetic Improvement Scale (GAIS), recorded at 90 days. This patient‐reported outcome assesses overall aesthetic change compared to baseline on a 5‐point scale ranging from −2 = much worse, −1 = worse, 0 = no change, +1 = improved, to +2 = much improved.

Safety was monitored by registering adverse events throughout the study period. Patients were instructed to report any local or systemic reactions not only during follow‐up visits but also via direct mobile messaging, ensuring continuous monitoring and early detection of potential complications.

### 2.6 Statistical Analysis

All statistical analyses were conducted using Jamovi software (version 2.3.28, The Jamovi Project, Sydney, Australia) with significance level set at *p* ≤ 0.05 for all tests.

Descriptive statistics included means and standard deviations for continuous variables with normal distribution and medians with interquartile ranges for ordinal outcomes. Categorical variables, such as Fitzpatrick phototype and glabellar contraction patterns, were expressed as absolute frequencies and percentages.

Differences between contraction pattern groups at baseline, 15 days and 90 days after the treatment were assessed using the ANOVA and Kruskal–Wallis test for continuous and ordinal variables respectively (age, BMI, GAIS, and GLSS).

Changes in glabellar line severity over time (baseline, 15 days, and 90 days) were evaluated with the Wilcoxon signed rank test with time as the within‐subject factor, and the Kruskal–Wallis test for contraction pattern as the between‐subject factor.

To identify predictors of treatment response, a multivariate ordinal logistic regression was performed at 15 days and 90 days, with GLSS as the dependent variable and age, BMI, Fitzpatrick phototype, and contraction pattern as independent variables. Model fit was reported using deviance, AIC, and McFadden's pseudo‐*R* ^2^ values.

## 3 Results

### 3.1 Demographics

**FIGURE 3:**
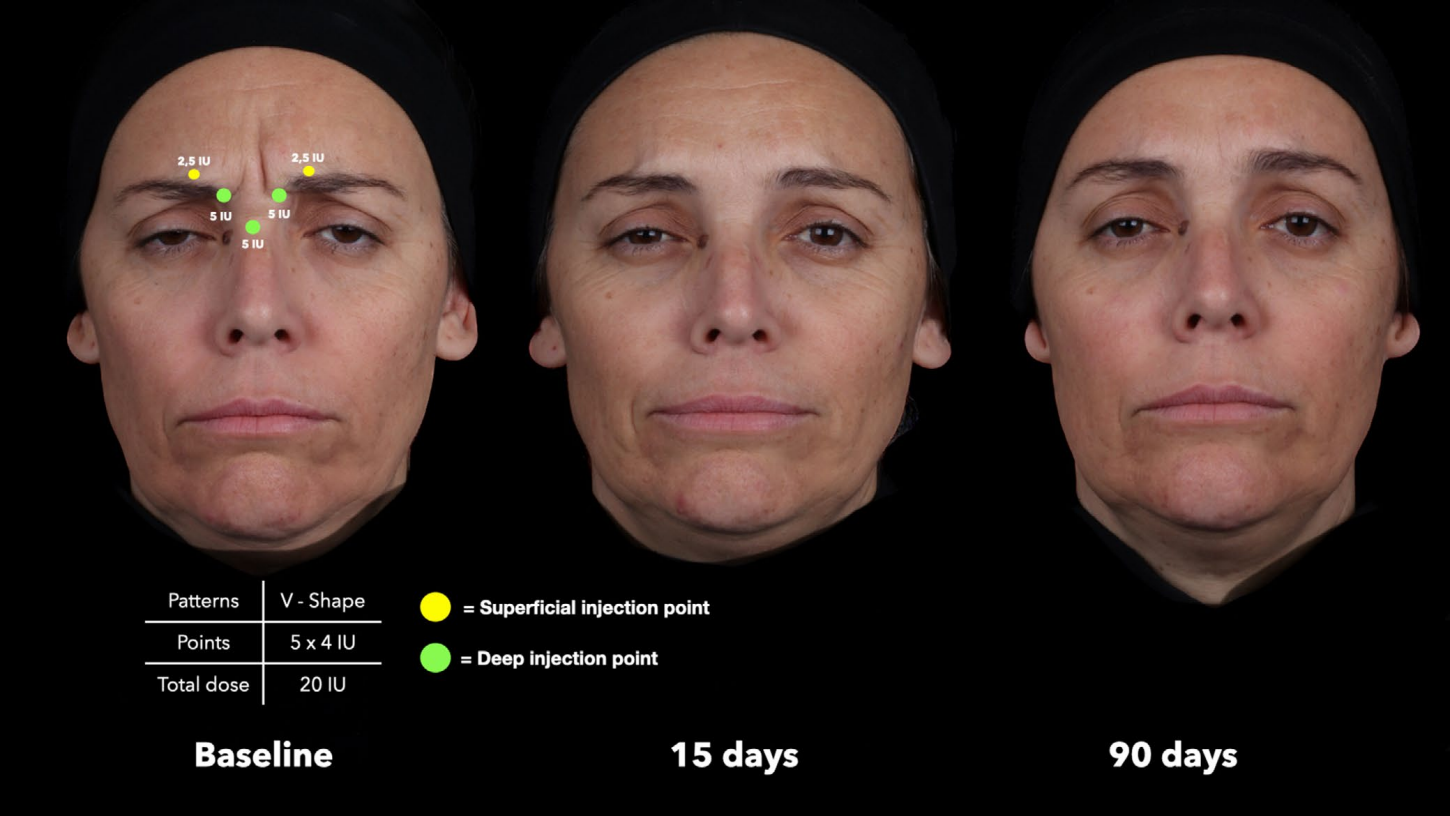
Representative treatment sequence of a 52‐year‐old female patient with V‐Shape contraction pattern. Assessments at baseline, 15 days, and 90 days after treatment show marked improvement in glabellar line severity.

**FIGURE 4:**
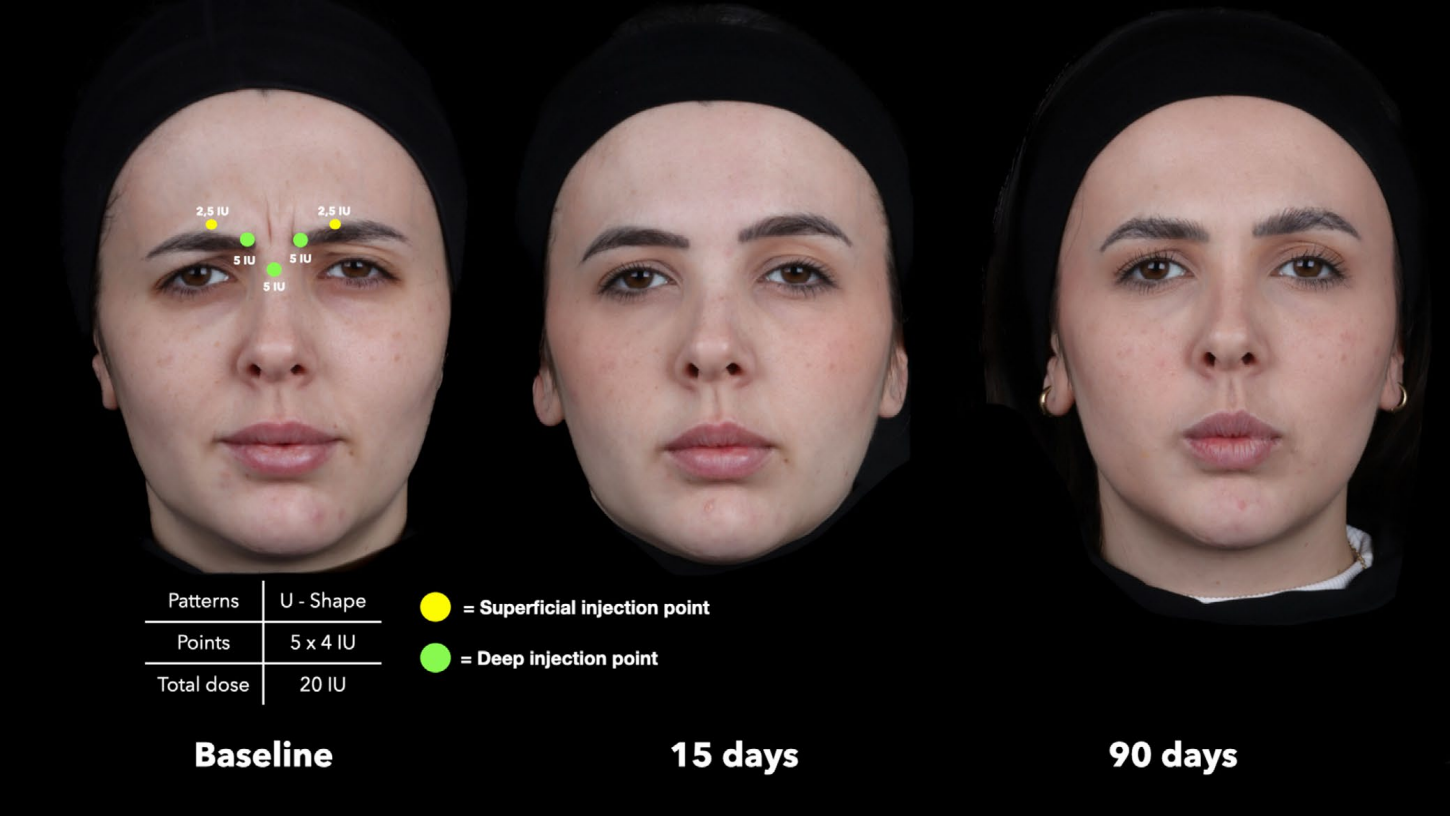
Representative treatment sequence of a 34‐year‐old female patient with U‐Shape contraction pattern. Assessments at baseline, 15 days, and 90 days after treatment show marked improvement in glabellar line severity.

**FIGURE 5:**
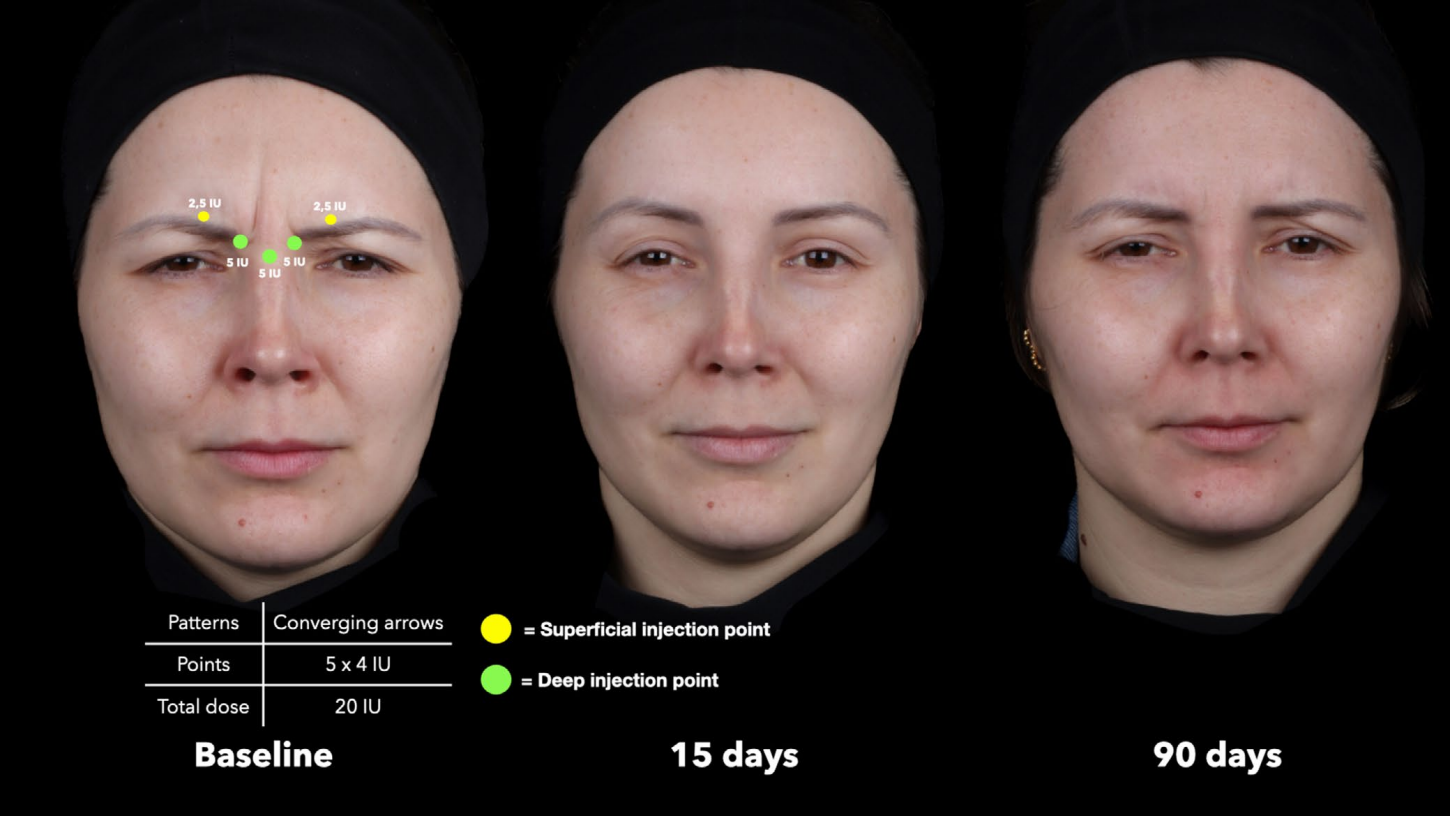
Representative treatment sequence of a 42‐year‐old female patient with Converging arrows contraction pattern. Assessments at baseline, 15 days, and 90 days after treatment show marked improvement in glabellar line severity.

Of the 125 patients enrolled, 119 (12 male and 107 female) completed the study protocol and were included in the final analysis. The mean age of participants was 40.4 ± 10.5 years, and the mean body mass index (BMI) was 26.6 ± 4.9 kg/m^2^ at baseline. The median baseline GLSS was 2.0 (IQR = 1.0). Fitzpatrick skin types ranged from I to VI, with the majority being type III (*n* = 37, 31.1%) and type IV (*n* = 33, 27.7%). Patients were classified into five glabellar contraction patterns: V‐shape (*n* = 16, 13.4%), U‐shape (*n* = 40, 33.6%), Converging Arrows (*n* = 36, 30.3%), Inverted Omega (*n* = 13, 10.9%), and Omega (*n* = 14, 11.8%) (Figure 2). No statistically significant differences were observed across contraction pattern groups for age (*p* = 0.553), BMI (*p* = 0.906), or baseline GLSS (*p* = 0.058) (Figures 3, 4, 5).

### 3.2 Glabellar Severity and Aesthetic Perception After Treatment

**FIGURE 6:**
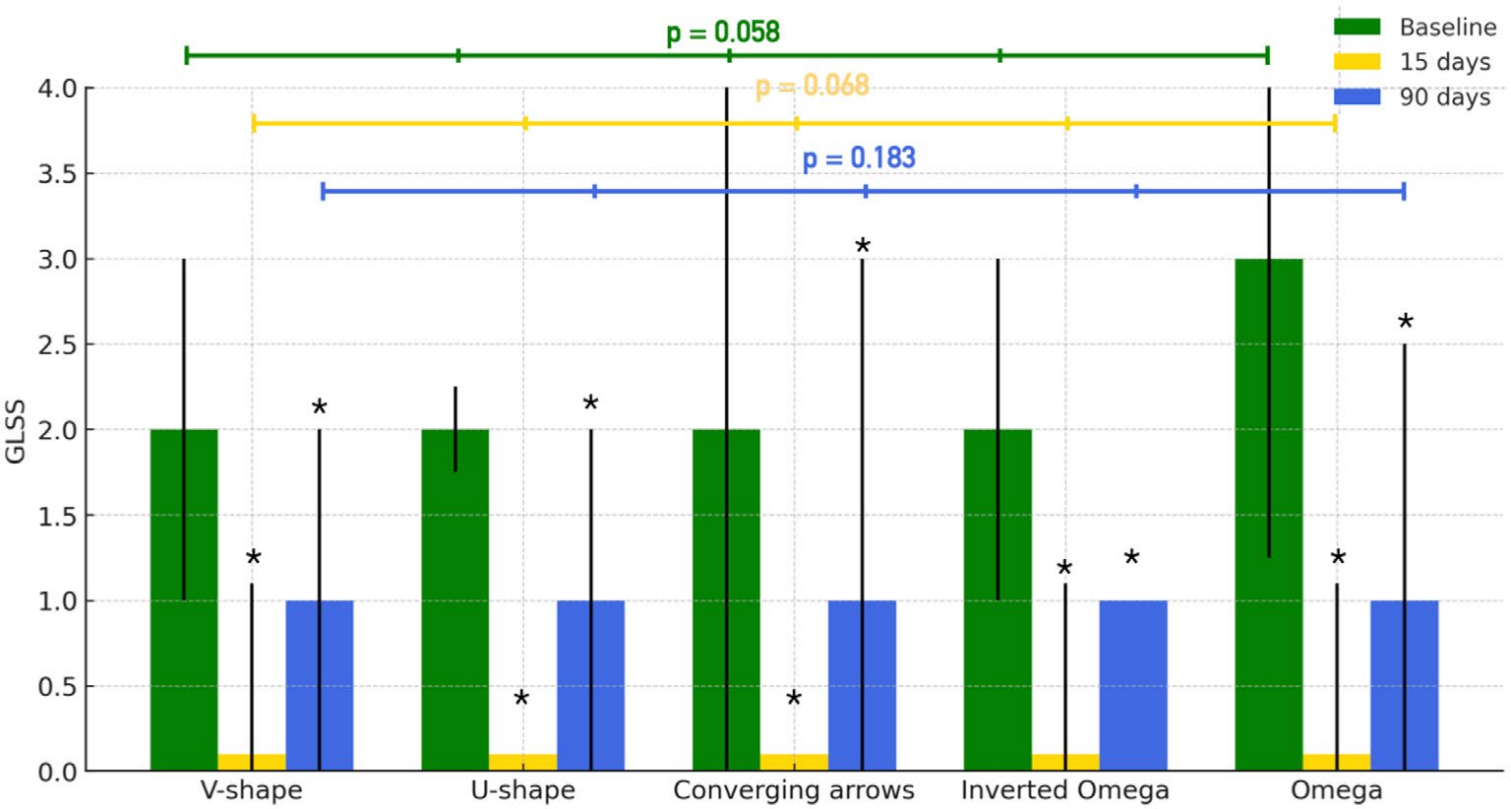
Median glabellar line severity score (GLSS) with interquartile range (IQR) at baseline, 15 days, and 90 days after neuromodulator treatment, stratified by glabellar contraction patterns (V‐shape, U‐shape, converging arrows, inverted omega, and omega). Statistically significant reductions compared with baseline are indicated by asterisks (*).

At baseline, the median GLSS for the entire sample was 2.0 (IQR = 1.0). A marked reduction was observed at 15 days post‐treatment, with a median GLSS of 0.0 (IQR = 1.0; *p* < 0.001), followed by a slight increase at 90 days with a median score of 1.0 (IQR = 1.0; *p* < 0.001 vs baseline and 15 days) (Figures 3, 4, 5, 6). Comparing the treatment outcomes across the five different glabellar contraction pattern groups showed that no statistically significant differences were noted at 15 days post‐treatment (*p* = 0.068) or at 90 days post‐treatment (*p* = 0.183) (Figure 6).

The GAIS evaluation for the entire cohort showed a median score of 1.00 (IQR = 1.0), corresponding to a perception of improvement to much improvement. When analyzed by contraction pattern, median values were 1.00 (IQR = 0.0) for V‐shape, 1.00 (IQR = 1.0) for U‐shape, 1.00 (IQR = 1.25) for Converging Arrows, 1.00 (IQR = 1.0) for Inverted Omega, and 1.00 (IQR = 2.0) for Omega, with no statistically significant differences among them (*p* = 0.479).

### 3.3 Multivariate Analysis of Treatment Outcome

Ordinal logistic regression analysis at 15 days post‐treatment identified age as a significant predictor of GLSS outcomes (*p* = 0.008), indicating that older patients were more likely to maintain higher severity scores. BMI showed a non‐significant trend toward improved outcomes (*p* = 0.099), while all Fitzpatrick types and glabellar contraction patterns did not significantly influence the results (all *p* > 0.05).

At 90 days, age (*p* < 0.001) and BMI (*p* = 0.003) emerged as significant predictors of GLSS, with older patients exhibiting higher severity scores and individuals with higher BMI tending to sustain greater improvements. Moreover, no significant effects were found for Fitzpatrick type or any of the glabellar contraction patterns (all *p* > 0.05).

### 3.4 Product Differences

No statistically significant differences in the GLSS or in the GAIS at 15 or at 90 days were observed when stratifying the study sample by product utilized (onabotulinumtoxinA vs. abobotulinumtoxinA), with all *p* > 0.05.

### 3.5 Adverse Events

Mild and transient local adverse events were observed in a small number of patients, including localized edema and hematoma at the injection sites. All reported events resolved spontaneously without the need for medical intervention, and no severe or unexpected adverse events were recorded during the study period.

## 4 Discussion

The present prospective, multicenter clinical study investigated whether different glabellar contraction patterns influence the clinical outcome of neuromodulator injections when a standardized and FDA‐approved 5‐point injection algorithm is applied. A total of 119 randomly selected patients were treated with the same 5‐point injection algorithm independent of their baseline glabellar contraction pattern. The results revealed that, despite clear differences in skin surface rhytid patterns existing at baseline, treatment efficacy, as assessed by both objective (GLSS) and subjective (GAIS) measures, was not statistically significantly different between glabellar contraction patterns at either early (15 days) or later (90 days) follow‐up time points.

These findings are highly consistent with previous clinical and anatomical evidence questioning the clinical relevance of contraction‐pattern–driven treatment algorithms. In particular, the results corroborate those reported by Germani et al. (2025), who demonstrated in a retrospective study design that identical glabellar injections yielded comparable outcomes across all five contraction patterns when the novel 3‐point injection technique was deployed [8].

From a clinical perspective, the absence of differences in clinical outcome between contraction patterns supports the notion that surface wrinkle morphology does not reliably reflect meaningful variations in the morphology or function of the glabellar muscles. This interpretation aligns with recent MRI‐based findings, which revealed no statistically significant differences in the length, thickness, or width of the procerus, corrugator supercilii, orbicularis oculi, or frontalis muscles when stratified by contraction pattern [5]. Thus, it appears that the neuromuscular substrate targeted by botulinum toxin remains largely consistent, irrespective of the visible skin pattern produced during contraction.

An important contribution of the present study is its prospective and multicenter design, which strengthens the external validity of these observations. Unlike earlier retrospective analyses with limited sample sizes (*n* = 42) [8], the current investigation included a larger, multi‐ethnic cohort treated across five independent centers. The consistency of outcomes across centers further supports the robustness of the findings and reinforces their relevance to real‐world clinical practice.

Another clinically relevant observation relates to the temporal behavior of treatment efficacy. As expected, a marked improvement in glabellar severity was observed at 15 days, followed by partial regression at 90 days, without pattern‐specific differences. This temporal profile reflects the known pharmacodynamics of botulinum toxin at the neuromuscular junction [11, 12] and suggests that contraction pattern does not influence either the onset or short‐term persistence of clinical effect when standardized doses and injection depths are used.

The multivariate analysis conducted provides additional insight into factors influencing treatment outcomes. While glabellar contraction patterns and Fitzpatrick skin type were not predictive of GLSS scores at either follow‐up point, age emerged as a significant predictor at both 15 and 90 days, with older patients exhibiting higher residual severity scores. This finding is biologically plausible and likely reflects age‐related changes in skin elasticity, connective tissue integrity, and dermal thickness, which are not directly addressed by neuromodulation alone. Interestingly, BMI showed a positive association with sustained improvement at 90 days, suggesting that subcutaneous tissue thickness can modulate the visible rhytid severity following a neuromodulator treatment over time [13].

The findings of the present study have direct clinical implications. Modifying injection points or dose distribution solely based on skin surface rhytids may be unnecessary and potentially misleading. Instead, an anatomy‐driven approach that respects known muscle origins, insertions, and depth relationships appears sufficient to achieve consistent and safe clinical outcomes [6, 14]. Overreliance on surface wrinkle patterns could, in contrast, increase the risk of suboptimal efficacy or adverse events, particularly if injections deviate from established anatomical safe zones [15].

The standardized five‐point injection protocol used in this study also deserves consideration. By addressing both the origin and insertion of the corrugator supercilii as well as the procerus muscle, this approach ensures balanced neuromodulation of the primary depressor complex of the glabella [16]. The absence of significant adverse events across a large sample further supports the safety of this technique when performed by experienced practitioners [14].

Limitations should be acknowledged. Although the study included a larger cohort than most previous investigations, longer follow‐up periods would be required to evaluate potential differences in duration of effect beyond 90 days. Additionally, while two different botulinum toxin formulations were used with a standardized conversion ratio, subtle pharmacological differences between products cannot be entirely excluded. Future studies might also explore whether dose escalation or alternative injection techniques yield similar pattern‐independent outcomes.

## 5 Conclusion

The present study demonstrates that glabellar contraction patterns do not significantly influence clinical or patient‐reported outcomes when neuromodulator injections are performed using a standardized, anatomy‐based, FDA‐approved 5‐point injection algorithm. Importantly, age and body mass index can be considered as relevant modifiers of treatment response over time, with increasing age associated with higher residual glabellar severity and higher BMI showing a tendency toward more sustained improvement. Together, these findings reinforce that glabellar neuromodulation should prioritize anatomical principles and patient‐specific biological factors, such as age and BMI, rather than skin surface contraction patterns, supporting a more reproducible, evidence‐based, and clinically applicable treatment paradigm.

## Author Contributions

M.G., V.R.M.M.‐L., and S.C. contributed to the conception and design of the study, participated in all phases of the research, and were actively involved in data analysis and interpretation. M.G., V.R.M.M.‐L., and S.C. wrote the manuscript. A.M.G., H.H., L.I., S.V., V.B., V.T., and K.D. contributed to data acquisition and execution of the study procedures. M.H.G., C.B., S.E., and R.R. provided critical revision of the manuscript for important intellectual content. All authors reviewed, read, and approved the final version of the manuscript.

## Funding

The authors have nothing to report.

## Consent

Written informed consent was obtained from all participants prior to their inclusion in the study and for the publication of clinical photographs.

## Conflicts of Interest

The authors declare no conflicts of interest.

## Data Availability

The data that support the findings of this study are available from the corresponding author upon reasonable request.
